# Adrenomedullin Contributes to Age-Related Memory Loss in Mice and Is Elevated in Aging Human Brains

**DOI:** 10.3389/fnmol.2017.00384

**Published:** 2017-11-15

**Authors:** Ignacio M. Larrayoz, Hilda Ferrero, Eva Martisova, Francisco J. Gil-Bea, María J. Ramírez, Alfredo Martínez

**Affiliations:** ^1^Oncology Area, Center for Biomedical Research of La Rioja (CIBIR), Logroño, Spain; ^2^Department of Pharmacology and Toxicology, University of Navarra, Pamplona, Spain

**Keywords:** adrenomedullin, normal aging, Alzheimer’s disease, memory loss, microtubules, p-Tau

## Abstract

Memory decline is common in elderly individuals and is the hallmark of Alzheimer’s disease (AD). Memory failure follows the loss of synaptic contacts in the cerebral cortex and hippocampus, caused in part by cytoskeleton disruption. Adrenomedullin (AM) and its gene-related peptide, proadrenomedullin N-terminal 20 peptide (PAMP), are microtubule-associated proteins (MAP) whose expression has been identified as a potential biomarker for predicting progression from predementia to clinical AD. Here we analyze the connection between AM levels and memory preservation. Mice lacking neuronal AM and PAMP (knockout, KO) and their wild type (WT) littermates were subjected, at different ages, to the novel object recognition test and the contextual fear conditioned test. Aged KO mice have significantly better retention memory than their WT counterparts. This feature was more prominent in females than in males. Prefrontal cortex and hippocampus samples from these animals were subjected to Western blotting for phospho-Tau and acetylated tubulin. Aged female KO mice had significantly less accumulation of phospho-Tau than their WT littermates. In addition, protein extracts from the frontal cortex of non-demented mature (65.10 ± 3.86 years) and aged (77.14 ± 2.77 years) human donors were analyzed by Western blotting. Aged human brains had significantly higher levels of AM and lower levels of acetylated tubulin than younger donors. These observations suggest that drugs or interventions that reduce AM/PAMP expression may constitute a new avenue to prevent memory decline during normal aging and in patients suffering moderate AD in high risk of rapid cognitive decline.

## Introduction

Memory loss is a common characteristic of normal aging (Leal et al., 2017) which gets greatly accelerated in some neurodegenerative diseases. Alzheimer’s disease (AD) is the most frequent cause of memory loss and other dementia symptoms among elderly patients (Hickman et al., 2016). AD is an irreversible degenerative pathology of the brain characterized by progressive deterioration of cognitive functions, affecting mainly neurons in the hippocampus and the cerebral cortex. The histological hallmarks of AD include senile plaques, made up by accumulations of β-amyloid (Aβ) peptide, and neurofibrillary tangles, which are deposits of Tau, a microtubule associated protein (MAP), which gets abnormally phosphorylated (pTau; Hernandez et al., 2013). Pathological accumulation of pTau, which compromises synaptic transmission and neuronal viability by contributing to cytoskeleton collapse, correlates better with the severity of cognitive decline in AD than senile plaques (Nelson et al., 2012).

The causes of memory loss during normal aging are not completely understood. Atrophy of some brain areas has been shown in normal aging (Pini et al., 2016) and changes in intrinsic neural electrical excitability associated with oxidative stress have been hypothesized as potential causes (Hermann et al., 2014). Subtle perturbations in stabilization of neuronal cytoskeleton, reminiscent of those occurring during AD neurodegeneration, may also be an important underlying cause of age-associated neuronal dysfunction and cognitive decline (Savva et al., 2009). In this line, modifications on pTau expression and status (tauopathies) are also typical of normal aging (Delacourte et al., 2002) and their distribution pattern correlates with memory capabilities (Guillozet et al., 2003).

Some interventions have been proposed to delay aging-related cognitive deficits and thus improve the quality of life in older people. Some of these interventions include mental stimulation and physical activity (Collette and Salmon, 2014) but a better knowledge of the mechanisms underlying the loss of memory may help in devising novel pharmaceutical approaches.

In the search for predictive blood biomarkers of AD cognitive decline, some studies have found that mid-regional proadrenomedullin is elevated in the plasma of AD patients and that the concentration of this peptide could have predictive value in the progression from predementia to clinical AD (Buerger et al., 2011; Henriksen et al., 2014), although a recent study on a Swedish population found no correlation (Holm et al., 2017). The proadrenomedullin gene, *adm*, codes for a 185 amino acid preprohormone which, after post-translational modifications, generates two biologically active peptides: proadrenomedullin N-terminal 20 peptide (PAMP) and adrenomedullin (AM). Both peptides are amidated at their carboxy terminus and their 3-dimensional structure is based on a central α-helix (Pérez-Castells et al., 2012). Expression of these peptides is widespread and several functions have been ascribed to them, including vasodilatation, bronchodilatation, angiogenesis, hormone secretion regulation, growth modulation and antimicrobial activities, among others (López and Martínez, 2002). In the central nervous system (CNS), AM is expressed throughout the whole brain and spinal cord (Serrano et al., 2000) where it acts as a neuromodulator through mechanisms dependent and independent of NMDA receptors (Xu and Krukoff, 2004). It has been shown that plasma levels of AM increase with normal aging (Kato et al., 2002).

Knockout studies have shown that total abrogation of *adm* results in embryo lethality (Caron and Smithies, 2001). To circumvent this problem, we generated a conditional knockout model where *adm* was eliminated just from neurons by using Cre/loxP technology, and the physiological consequences of such manipulation have been published (Fernández et al., 2008, 2010; Hurtado et al., 2010).

An intriguing finding from our laboratory showed that both AM and PAMP decorate the microtubules in a variety of cell types, including neurons (Sackett et al., 2008). Yeast-2-hybrid analysis demonstrated that AM binds to several MAPs, whereas PAMP binds directly to tubulin and kinesin. Cell physiology studies point to a direct involvement of PAMP in regulating microtubule dynamics and kinesin speed (Larráyoz and Martínez, 2012). Downregulation of *adm* expression, through either gene knockdown or targeted knockout, results in a massive hyperpolymerization of the tubulin cytoskeleton, an increase on Glu- and acetylated-tubulin, a reduction of kinesin velocity, and the apparition of actin filopodia in CNS stem/progenitor cells (Sackett et al., 2008; Vergaño-Vera et al., 2010). In addition, AM immunoreactivity increases in the brain of AD patients (Ferrero et al., 2017) and in mouse models of AD where it seems to be associated with activated astrocytes in the vicinity of amyloid plaques (Fernandez et al., 2016).

Taking all this into consideration we decided to investigate the potential connection between *adm* gene products and normal aging memory loss in a mouse model and the expression of AM in human brains.

## Materials and Methods

### Knockout (KO) Mice Lacking Neuronal AM

Conditional knockout (KO) mice where AM was eliminated from neurons have been previously described (Fernández et al., 2008). KO and wild type (WT) littermates of both sexes were allowed free access to food and water under standard laboratory conditions, with light/dark cycles of 12/12 h, and a constant temperature of 24ºC. All procedures were carried out in accordance with the European Communities Council Directive (86/609/CEE) on animal experiments and with approval from the ethical committees on animal welfare of our institutions (OEBA-CIBIR and University of Navarra protocol 054-12).

### Behavioral Tests

#### Novel Object Recognition Test (NORT)

Test was performed as previously described (Gerenu et al., 2013) in young (3 months old) and old (18 months old) mice. In short, animals (*n* = 8 per group) were first familiarized with the arena (65 × 65 × 45 cm) for 30 min and after 1 day mice were allowed to explore two identical objects during 5 min (training). Retention was assessed 24 h post-training, when one object was replaced by a novel one. Retention score is expressed as discrimination index (percentage of time exploring the novel object to the total time of object exploration).

#### Contextual Fear Conditioned Test

After 24 h of rest, all mice were subjected to a fear conditioned test. The behavioral procedure involved three phases: habituation, training, and testing. Mice were habituated for 5 min to the context, which consisted in a soundproof box with white walls, light, and a background noise produced by a fan. The training phase was conducted 24 h later, where mice were placed in the same context and allowed to explore for 2 min prior to a 2-s footshock (0.3 mA) stimulus. After 30 s mice were returned to their home cage. 24 h later mice were placed back in the conditioning box and allowed to explore the context for 2 min, during which freezing time was recorded (contextual long-term memory). Freezing behavior was defined as an absence of cage displacement. Freezing scores were expressed as percentages of total freezing time. The conditioning procedure was carried out in a StartFear system (Panlab S.L., Barcelona, Spain) that allows movement recording by a high-sensitivity Weight Transducer system and data analysis by the built-in FREEZING and STARTLE software.

### Human Brain Tissue

Brain tissues were obtained from the Oxford Project to Investigate Memory and Ageing (OPTIMA, see www.medsci.ox.ac.uk/optima). Subjects for this study constituted a randomly selected subset of the participants, now part of the Thomas Willis Oxford Brain Collection within the Brains for Dementia Research Initiative (BDR). At death, informed consent had been obtained from the patients’ next-of-kin before collection of brains and the study was approved by the UK National Research Ethics Service. All cases were selected based on clinic-pathological consensus diagnoses. A total of 12 individuals were included in the study. They included six mature donors (3 men, 3 women, age at death = 65.10 ± 3.86 years; post mortem delay = 34.10 ± 4.91 h) and six older donors (3 men, 3 women, age at death = 77.14 ± 2.77 years; post mortem delay = 49.42 ± 6.82 h). These participants were classified as normal controls, did not have dementia or other neurological diseases, did not meet CERAD criteria for AD diagnosis, and were staged at Braak 0-II. Frontal (Brodmann Area, BA10) cortices were dissected free of meninges. To partially mitigate the possible effects of cause of death on neurochemical determinations, brain pH was measured as an index of acidosis associated with terminal coma. Brain pH is used as an indication of tissue quality in post-mortem research, with pH > 6.1 considered acceptable (Bahn et al., 2001; Lewis, 2002). All the tissue used fulfilled this condition. All subsequent analyses were performed blind to clinical information.

### Western Blotting

Two days after completing behavioral testing, all mice were euthanized and prefrontal cortex and hippocampus were dissected out. Mouse and human samples were homogenized in RIPA buffer (Thermo Scientific, Rockford, IL, USA) containing protease (EDTA-free complete, Roche, Basilea, Switzerland) and phosphatase (PhosStop, Roche) inhibitors. Homogenates were centrifuged for 30 min at 15,000× *g* and the supernatants collected. Protein concentration was determined by the BCA kit (Pierce, Rockford, IL, USA), with bovine serum albumin as standard, using a spectrophotometer (POLARstar Omega, BMG Labtech, Ortenberg, Germany). Then, 25 μg of each sample were mixed with 4× sample buffer (Invitrogen, Carlsbad, CA, USA) and heated for 10 min at 70°C. Samples were run on 4%–12% SDS–polyacrylamide gels. Seeblue plus 2 Prestained Standards (Invitrogen) were used as molecular weight markers. Proteins were transferred onto 0.2-μm nitrocellulose membranes (Amersham GE HealthCare, Pittsburgh, PA, USA). Membranes were incubated overnight at 4°C with primary antibodies followed by peroxidase-labeled secondary antibodies (Table 1). Immunoreactive bands were visualized using enhanced chemiluminescence and quantified by an image analyzer (Quantity One, Bio-Rad, Hercules, CA, USA). Membranes were stripped with Restore PLUS Western Blot Stripping Buffer (Thermo Scientific). β-Actin was used as an internal loading control. Results were calculated as the percentage of optical density (OD) values of the WT.

**Table 1:** Antibodies and conditions used in this study.

Target	Species	Dilution	Reference
Adrenomedullin	Rabbit polyclonal	1:500	Novus NBP1-19731
AT8	Mouse monoclonal	1:1000	Thermo Scientific MN1020
PHF1	Mouse monoclonal	1:1000	Gift from Peter Davies
TAU	Mouse monoclonal	1:1000	Cell Signaling 4019
Acetylated tubulin	Mouse monoclonal	1:15,000	Sigma T7451
β-Actin	Mouse monoclonal	1:10,000	Sigma AC74
**Secondary peroxidase-labeled antibodies**			
**Specificity**	**Host**	**Dilution**	**Reference**
Anti-mouse	Goat	1:5000	Dako P0447
Anti-rabbit	Goat	1:5000	Dako P0448

### Statistical Analysis

Data were analyzed by SPSS for Windows, release 15.0. Normalcy and homoscedasticity were checked by Shapiro–Wilks’s and Levene’s tests, respectively. Normally distributed data were analyzed by Student’s *t*-test or two-way analysis of variance (ANOVA) (genotype × age) followed by Tukey’s *post hoc* test. *P* values lower than 0.05 were considered statistically significant.

## Results

### Mouse Experiments

The conditional AM KO animals and their WT littermates were tested in two different cognitive paradigms: the novel object recognition test (NORT) and the fear conditioning test. Animals included were either young (3 months) or aged (18 months) mice. Each age group was composed by KO (*n* = 8) and WT (*n* = 8) littermates.

**Figure 1:**
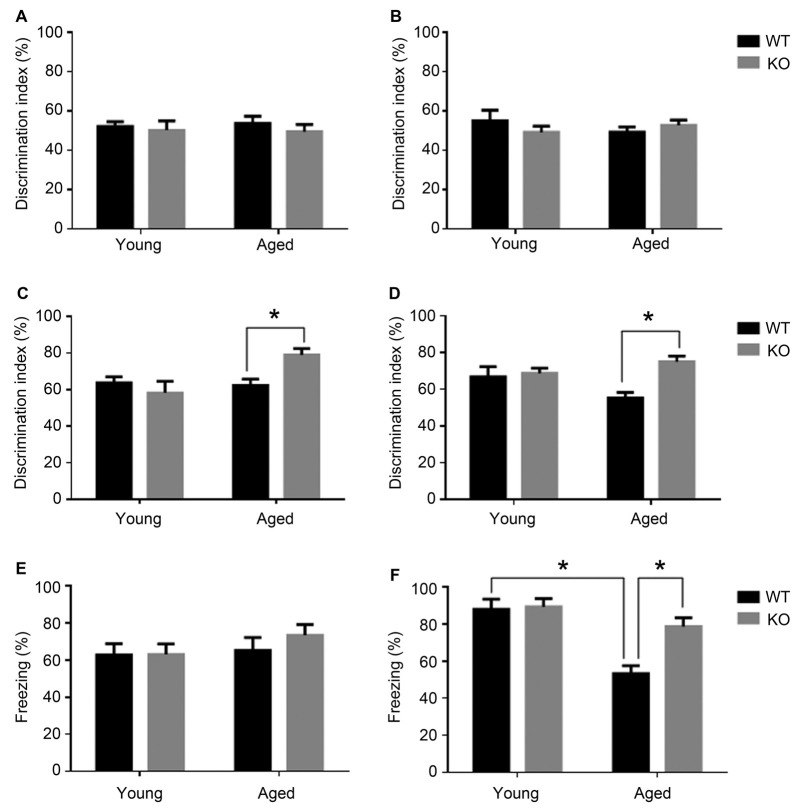
Cognitive phenotype in animals lacking neuronal *adm* as tested in the novel object recognition test (NORT, **A–D**) and fear conditioning **(E,F)**. Behavioral NORT data are shown as percentage of discrimination index (time exploring new object/total time of exploration *100) in male **(A,C)** and female **(B,D)**, young (3 months) and aged (18 months) mice. Discrimination index was measured with two identical objects on day 2 **(A,B)** and after introducing the novel object on day 3 **(C,D)**. Fear conditioning data are shown as percentage of freezing over the 2 min test in male **(E)** and female **(F)** young and aged mice. Two-way analysis of variance (ANOVA) (age × genotype), *p < 0.05 interaction. WT, wild type; KO, *adm* knockout.

First of all, to ensure that WT and KO mice had no differences in general locomotor activity or other psychological/physiological traits that may interfere with memory determination, their investigative activity was recorded on the first and second day of the experiment. On the first day, in the first 30 min, WT mice traveled 18871 ± 2362 cm whereas KO mice walked for 16486 ± 1666 cm (*t* test, *p* > 0.05). On the second day (training), when mice were presented with two identical objects, all groups of mice had a ratio close to 50% and there were no significant differences among them (*t* test, *p* > 0.05; Figures 1A,B). These results show that genotype does not influence mice ability to move freely or to show curiosity when presented with different objects.

When presented with the novel object, young animals had no significant differences in recognition memory between those carrying *adm* and the knockouts (Figures 1C,D). Recognition memory was significantly facilitated in aged *adm* KO animals, both in males (Figure 1C, two way ANOVA, significant interaction *F* = 12.44, *p* < 0.05, Tukey’s multiple comparisons test, *p* < 0.05) and females (Figure 1D, two way ANOVA, significant interaction *F* = 11.11, *p* < 0.05, Tukey’s multiple comparisons test, *p* < 0.05).

In the fear conditioning test (contextual learning), first we measured the freezing levels during training and found they were around 40% for both genotypes (*t* test, *p* > 0.05) during the first 2.5 min of their stay in the chamber. We also tested foot shock sensitivity by measuring the freezing levels immediately following the foot shock. These levels were close to 80% for both genotypes (*t* test, *p* > 0.05) for the 30 s following the shock, indicating that the genotype has no influence in regular freezing behavior or in foot pain sensitivity.

During the memory test period, male mice showed no statistically significant differences in freezing behavior irrespective of age or genotype (Figure 1E). In contrast, older female WT mice presented an age-related memory loss as demonstrated by a statistically significant reduction in freezing time when compared with younger females of the same genotype (Figure 1F, two way ANOVA, significant interaction *F* = 9.02, *p* < 0.05, Tukey’s multiple comparisons test, *p* < 0.05). Interestingly, old female mice lacking neuronal *adm* showed better memory than their WT littermates (Tukey’s multiple comparisons test *p* < 0.05).

Altogether, both cognitive tests point to a memory protection phenotype in animals lacking neuronal *adm*.

**Figure 2:**
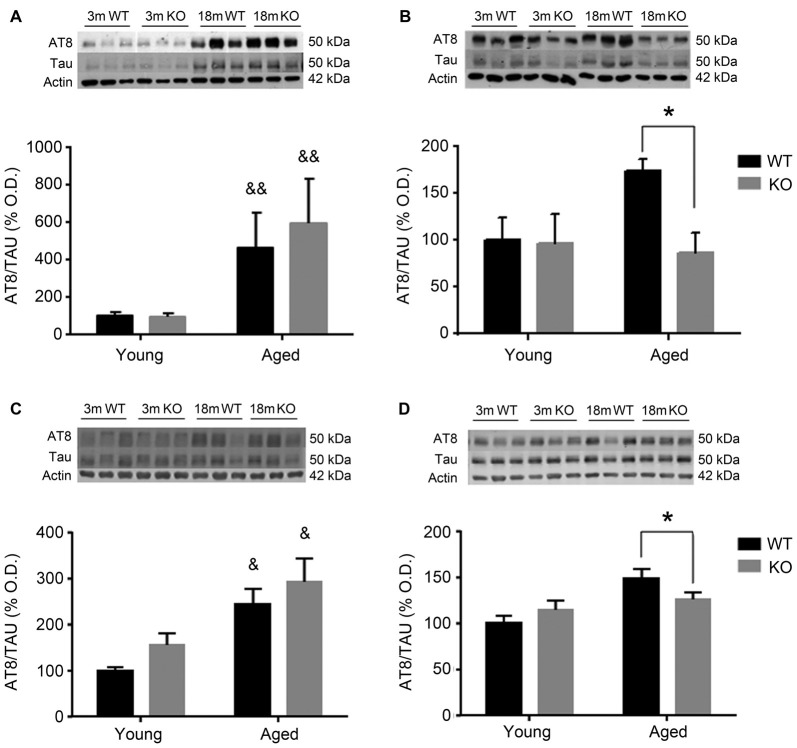
Expression of pTAU (using the AT8 antibody) in the frontal cortex **(A,B)** and the hippocampus **(C,D)** of male **(A,C)** and female **(B,D)**, young (3 months, 3 m) and aged (18 months, 18 m), control wild type (WT) and *adm* knockout (KO) mice. Two-way ANOVA: *p < 0.05 interaction, ^&^*p* < 0.05, ^&&^*p* < 0.01 main effect of age. Panels show percentage of optical density (OD) values of control and representative pictures of the blotting. β-Actin is used as internal loading control.

**Figure 3:**
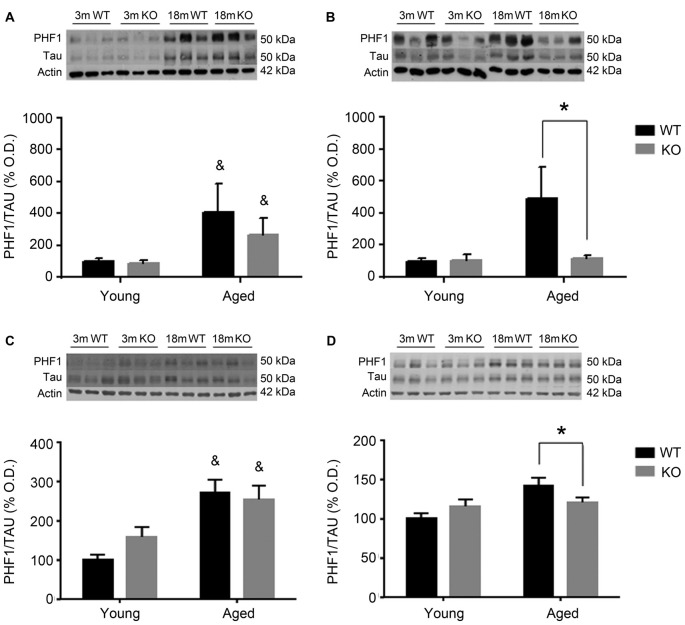
Expression of pTAU (using the PHF1 antibody) in the frontal cortex **(A,B)** and the hippocampus **(C,D)** of male **(A,C)** and female **(B,D)**, young (3 months, 3 m) and aged (18 months, 18 m), control (WT) and *adm* knockout (KO) mice. Two-way ANOVA: *p < 0.05 interaction, ^&^*p* < 0.05 main effect of age. Panels show percentage of OD values of control and representative pictures of the blotting. β-Actin is used as internal loading control. β-Actin and total TAU are the same as in Figure 2 and are repeated here to allow a direct comparison with the total protein and loading controls.

To investigate the possible mechanism underlying these behavioral observations, changes in pTau expression were checked in the mouse prefrontal cortex and hippocampus using two different antibodies: AT8 (Figure 2) and PHF1 (Figure 3). In male mice, there was an increased pTau/Tau ratio associated to aging that was not significantly affected by deleting the *adm* gene (two way ANOVA, main effect of age, *F* = 21.50, *p* < 0.01, Figures 2A,C). However, in female mice, a significant interaction between genotype and age was found (Figures 2B,D), and the increased expression of pTAU associated to aging was counteracted in mice lacking the *adm* gene (two way ANOVA, significant interaction *F* = 16.81, *p* < 0.05, Tukey’s multiple comparisons test, *p* < 0.05).

For the other antibody, PHF1 (Figure 3), results were perfectly parallel to those observed with AT8. There was an increased pTau/Tau ratio associated to aging in males that was not significantly affected by deleting the *adm* gene (two way ANOVA, main effect of age, *F* = 28.81, *p* < 0.05, Figures 3A,C). In females, a significant interaction between genotype and age was found (Figures 3B,D), and the increased expression of pTAU associated to aging was counteracted in mice lacking the *adm* gene (two way ANOVA, significant interaction *F* = 18.24, *p* < 0.05 Tukey’s multiple comparisons test, *p* < 0.05).

**Figure 4:**
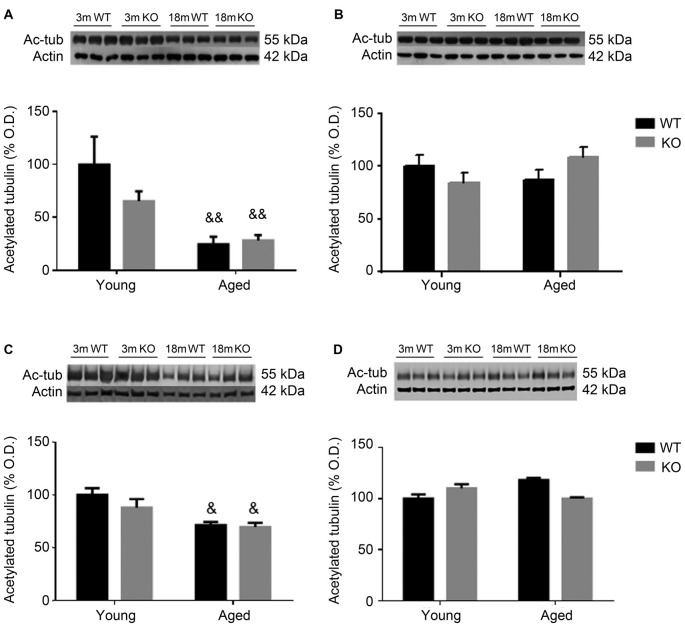
Expression of acetylated tubulin (Ac-tub) in the frontal cortex **(A,B)** and hippocampus **(C,D)** of male **(A,C)** and female **(B,D)**, young (3 months, 3 m) and aged (18 months, 18 m), control (WT) and *adm* knockout (KO) mice. Two-way ANOVA: ^&^*p* < 0.05 main effect of age, and ^&&^*p* < 0.01 main effect of age. Panels show percentage of OD values of control and representative pictures of the blotting. β-Actin is used as internal loading control.

To further study cytoskeleton stability, tubulin acetylation was checked in the frontal cortex and hippocampus of these animals. Aged male mice showed a decrease in the expression of acetylated tubulin that was independent of the genotype (two way ANOVA, main effect of age, *F* = 39.23, *p* < 0.01, Figures 4A,C). No effect associated to either age or genotype was found in female mice (Figures 4B,D).

### Human Specimens

**Figure 5:**
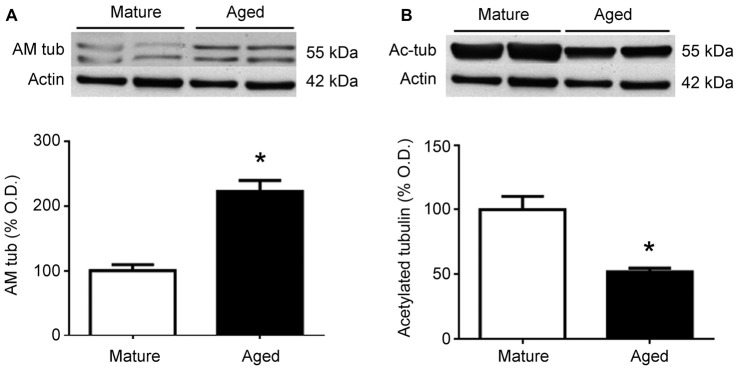
Expression of adrenomedullin (AM) **(A)** and acetylated tubulin **(B)** in the frontal cortex of mature and aged human donors. The band of immunoreactive tubulin-associated AM (~55 kDa) is more intense in the aged brain whereas acetylated tubulin is significantly lower in aged individuals. Student’s *t* test, *p < 0.05. β-Actin is used as internal loading control. β-Actin blot in **(B)** is the same as in **(A)** and is repeated here to allow a direct comparison with the loading controls.

To test whether similar changes happen in humans, frontal cortex protein extracts were obtained from mature and aged human donors. Western blots for AM showed a significant (*p* < 0.05) increase in the relative levels of this peptide in aged brains, which was more than double the levels observed in younger samples (Figure 5A). In contrast, the relative levels of acetylated tubulin were significantly (*p* < 0.05) reduced by aging (Figure 5B).

## Discussion

In this study we have shown that aged mice that lack neuronal AM have better contextual and recognition memory than their WT littermates. In parallel, the brain cortex and hippocampus of these mice have a lower accumulation of p-Tau, suggesting that p-Tau may be the link between lack of AM and memory preservation, although we cannot rule out other alternative molecular pathways. In addition, we also showed that older human individuals present higher levels of AM and lower levels of acetylated tubulin in their brains than younger controls.

Previous studies had found that plasma AM increases with age (Kato et al., 2002), but a description of the levels of AM in the aging brain was lacking. Here we have demonstrated that normal aging is accompanied by an increase of AM protein expression, at least in the frontal cortex. Previous reports have indicated that elevated AM levels occur in the brain of AD patients (Ferrero et al., 2017) and that plasma levels of the related peptide, mid-regional proadrenomedullin, may constitute an early marker for progression to AD (Buerger et al., 2011; Henriksen et al., 2014). Obviously, patients in their path to develop AD should have a larger AM increase than normally aging individuals. These correlation studies provide important information by identifying potential markers to detect early cases of rapidly declining individuals, but they do not help in understanding the underlying biochemical mechanisms. Our mouse study provides a first insight into the potential mechanisms linking AM levels to contextual memory loss, through the regulation of p-Tau, especially among females. Although a protein-protein contact between Tau and AM was not detected by yeast-2-hybrid experiments (Sackett et al., 2008), these two proteins are MAPs and are located in close proximity on the microtubule surface, so a direct physical interaction cannot be excluded. Alternatively, AM could modulate any of the kinases that phosphorylate Tau (Tell et al., 2016). The exact biochemical mechanism linking AM to Tau phosphorylation should be addressed in future studies.

Two different antibodies were used in this study to asses Tau phosphorylation. AT8 is a phospho-specific antibody that recognizes phosphorylation in Ser202 and Thr205 (Goedert et al., 1995), whereas the PHF1 antibody recognizes phosphorylation on Ser396 and Ser404 of the Tau protein (Otvos et al., 1994). All these phosphorylation sites have been involved in pathological findings (Mondragón-Rodríguez et al., 2014). PHF-1 is generally considered a later marker in the dynamic sequence of Tau phosphorylation events during the evolution of neurofilaments in AD, when compared to AT8 (Oh et al., 2010). Having very similar results with both antibodies indicates that we are detecting highly phosphorylated forms of Tau, which are the hallmarks of AD and other tauopathies related to memory loss (Tepper et al., 2014).

The levels of acetylated tubulin, a post-translational modification of α-tubulin which results in microtubule stability (Strzyz, 2016), were also studied as another potential mechanism of AM-mediated memory preservation. Aged human donors and male mice had lower levels than their younger counterparts, although this did not happen with female mice. In these animals we did not see any difference between genotypes, although we expected them based on previous studies (Sackett et al., 2008). Perhaps this loss of hyperpolymerized microtubules in the neurons occurs late in the aging process, resulting in synaptic disconnection, and is not so prevalent in females, where Tau phosphorylation may be more relevant. Of course, another possibility is that female hormones may have a neuroprotective effect in this context. Other α-tubulin modifications have been also reported to increase in neurodegenerative diseases, including AD (Vu et al., 2017), suggesting an important contribution of the cytoskeleton to the pathogenesis of memory loss.

An intriguing feature is the clear sexual dimorphism we observed in the behavioral and biochemical studies. Our fear conditioning results suggest that aged female mice are more prone to contextual memory loss than their male counterparts, a condition that was compensated by the lack of AM expression in neurons. Albeit using different memory tests, these sexual differences have been also reported in humans where women have an increased risk for memory disorders relative to men later in life (Jacobs et al., 2016; Reed et al., 2017). It seems that this risk depends on sex steroids, whose decline after menopause correlates with more pronounced alterations in hippocampal connectivity (Jacobs et al., 2016). In addition, the physiological effects of AM have shown to be also sex-dependent with females being more susceptible to changes in the levels of AM expression (Martínez-Herrero et al., 2016).

We have previously found that lack of AM has an impact on locomotor activity (Fernández et al., 2008) and pain perception (Fernández et al., 2010) in young mice. These characteristics may have an influence in the way the KO mice respond to the memory tests. To address these issues we performed an open field test in old mice before the NORT and found no differences in motility, as previously shown for mice of this age (Fernández et al., 2008). Since we do not observe any memory differences in younger mice and the older mice do not have locomotor activity differences, we can rule out the influence of this feature on the results of the NORT. Also, to ensure that the different sensitivity to pain did not influence the fear conditioning results, we measured the freezing behavior of mice of either genotype before and after the foot shock. In both cases, the differences were not statistically significant, indicating that this is not an issue for this specific test.

Previous studies have shown that neuronal AM may have a neuroprotective effect, especially in the context of stroke (Hurtado et al., 2010) and exposure to hypobaric environments (Fernández et al., 2008). Nevertheless, we cannot generalize and say that AM is always neuroprotective. For instance, high levels of circulating AM correlate with increased neurological severity in stroke patients (Serrano-Ponz et al., 2016) and endothelial AM seems to have the reverse effect on stroke and brain damage than neuronal AM (Ochoa-Callejero et al., 2016). Until we acquire a more complete understanding on the effects of AM in the CNS, our present data need to be interpreted on their own, indicating that lower levels of AM are beneficial for memory preservation, especially in females.

AM has been shown to bind to complement factor H (CFH) in the blood stream, and each molecule influences the physiological activities of the other (Pio et al., 2001). CFH is also present at high levels in the CNS (Serrano et al., 2003). Although there are no data on the evolution of brain CFH with age, some studies point to a correlation between lower levels of circulating CFH and more severe cognitive impairment during the development of AD, both in patients (Gezen-Ak et al., 2013) and in mouse models (Wang et al., 2016). It would be interesting to investigate the potential impact of CFH on AM-mediated memory loss during normal aging.

Our data suggest that reducing AM/PAMP levels may constitute a novel path to preventing/delaying memory loss. A few years ago, a particular single nucleotide polymorphism (SNP) close to the *adm* gene was found to be responsible for a natural reduction in the circulating levels of AM (Cheung et al., 2011) and to correlate with cancer susceptibility (Martínez-Herrero and Martínez, 2013). Therefore, it would be interesting to test whether carriers of this SNP are more protected from developing memory impairment. Also, several physiological inhibitors of AM have been proposed for clinical development, including a monoclonal antibody (Martínez et al., 1996), the peptide fragment AM_22–52_ (Ishikawa et al., 2003), and a number of small molecules that target either AM or PAMP (Martínez et al., 2004; Roldós et al., 2012). Therefore some of these inhibitors may be used for the pharmacological prevention of age-related memory loss.

In conclusion, normal aging results in higher expression of AM in the brain and AM ablation prevents Tau phosphorylation in female mice and favors memory preservation in advanced age. These observations suggest that a pharmacological inhibition of *adm* gene products may constitute a novel approach to preventing memory loss in normal aging and in patients suffering moderate AD in high risk of rapid cognitive decline.

## Author Contributions

AM and MJR conceived of the idea for the study. IML, HF, EM and FJG-B performed experiments and analyzed data. All authors agree to be accountable for all aspects of the work in ensuring that questions related to the accuracy or integrity of any part of the work are appropriately investigated and resolved. All authors contributed intellectually to the revision of the manuscript and approved the final version of the manuscript.

## Conflict of Interest Statement

The authors declare that the research was conducted in the absence of any commercial or financial relationships that could be construed as a potential conflict of interest.

## Abbreviations

AD: Alzheimer’s disease
AM: adrenomedullin
APP: amyloid precursor protein
CFH: complement factor H
CNS: central nervous system
KO: knockout
MAP: microtubule associated protein
NORT: novel object recognition test
PAMP: proadrenomedullin N-terminal 20 peptide
PHF: paired helical filaments
SNP: single nucleotide polymorphism
WT: wild type.
